# Editorial for the Special Issue on Micro- and Nanosensors: Fabrication, Applications, and Performance Enhancements, 2nd Edition

**DOI:** 10.3390/mi16060614

**Published:** 2025-05-24

**Authors:** Joanna Smajdor

**Affiliations:** Faculty of Materials Science and Ceramics, AGH University of Krakow, al. Mickiewicza 30, 30-059 Cracow, Poland; smajdorj@agh.edu.pl

Micro- and nanosensors are pivotal tools in various fields, revolutionizing the way in which we monitor and interact with biological, environmental, and industrial processes. Their fabrication, diverse applications, and innovative performance enhancements are fundamental to their advancement nowadays.

The fabrication of micro- and nanosensors involves advanced techniques that facilitate the creation of sensors with high sensitivity and specificity. Techniques such as electrochemical deposition and self-assembly are prevalent in the development of nanosensors [1]. Micro- and nanosensors can be made using a variety of materials, including silicon, polymers, and nanostructured metals or carbon-based materials. For instance, carbon quantum dots have shown potential in creating highly sensitive fluorescent nanosensors [2]. The integration of features such as biocompatibility and specificity through molecular imprinting has further enhanced the capabilities of these sensors, allowing for targeted applications such as real-time biochemical monitoring [3].

Micro- and nanosensors have found extensive applications across various domains. In biomedical applications, they are employed for the real-time monitoring of critical physiological parameters, such as glucose levels and pH, providing crucial data for managing diseases like diabetes [4,5]. In environmental monitoring, nanosensors detect contaminants and measure environmental parameters, playing an essential role in ensuring public safety and health [6]. For instance, novel nanosensors have been designed for the detection of explosives, achieving remarkable sensitivity [7]. Additionally, researchers have explored the use of nanosensors in agriculture, enabling the monitoring of soil moisture and nutrient levels to optimize crop production [8].

The performance of micro- and nanosensors can be significantly enhanced through various innovative strategies. The use of machine learning approaches has been shown to improve the detection limits of sensors, particularly in identifying heavy metals at low concentrations [2]. Moreover, incorporating advanced materials and designs, such as core–shell structures and hybrid nanomaterials, has allowed for the enhancement of sensitivity and response times [9]. For example, integrating nanomaterials with superior optical properties can lead to enhanced fluorescence signals, making it easier to detect the presence of specific analytes [10]. Enhancements in miniaturization and the development of self-powered sensors are further revolutionizing how these instruments function, making them more efficient and suitable for diverse applications [11].

This Special Issue consists of five papers, focused on the topics of the preparation, application, and performance of micro- and nanosensors, designed with the aim of crack detection and evaluation [12], current measurements [13], CO_2_ monitoring [14], curvature measurements [15], and low-temperature film design [16]. 

The first study by Fan et al. [12] introduces a differential tunnel magnetoresistance–alternating current field measurement (TMR-ACFM) probe that is integrated with a Convolutional Neural Network (CNN) and Convolutional Block Attention Module (CBAM), aimed at providing a quantitative evaluation of crack dimensions. Both theoretical and empirical data indicate that the design of the differential probe effectively mitigates the noise resulting from lift-off effects and external electromagnetic interference, which significantly improves sensitivity in detection. By utilizing a differential TMR bridge as the core sensing component, this probe maintains comparable manufacturing costs to conventional ACFM probes, achieving a more than tenfold enhancement in the quality factor of the differential signal and increasing the signal-to-noise ratio by over 3 dB. The output signal generated by the differential probe exhibits a clear relationship with the characteristics of the cracks, specifically their length, depth, and width. The length of the cracks is predominantly associated with the overall features of the output waveform, while the width and depth are primarily linked to the finer, localized attributes of the signal. By utilizing the experimental results gathered from the differential probe, the constructed CNN + CBAM network demonstrates an effective capability to predict crack dimensions with a level of accuracy that outperforms numerous alternative deep learning models.

Another paper by Xu et al. [13] details the design and performance assessment of a tunneling magnetoresistance (TMR) current sensor that offers high precision and resolution, specifically engineered for the detection of weak currents. The development process is comprehensively described and includes an analysis of the experimental principles, design of the sensor architecture, selection of the chip, design of the magnetic flux concentrator, and the creation of a corresponding testing system. The proposed sensor boasts several significant advantages, including an extensive measurement range, exceptional accuracy, high resolution, and the ability to perform non-invasive measurements. In the realm of chip selection, this sensor design incorporates a low-noise, low-hysteresis TMR chip. Furthermore, the sensor circuit employs a high-linearity interface circuit to mitigate the fixed bias, while the magnetic flux concentrator enhances sensitivity and resistance to interference. 

The study by Mota et al. [16] is based on the silicon (Si) usage in the preparation of low-temperature films for sensor applications. Polycrystalline silicon (poly-Si) films are synthesized on both silicon wafers and polymer substrates through a metal-induced crystallization (MIC) process facilitated by AlSiCu at a temperature of 450 °C for a duration of 8 h. The process is controlled via X-ray diffraction (XRD) analyses performed on the types of samples, checking the obtained crystallite sizes of the resultant poly-Si films. Scanning electron microscopy (SEM) examinations with a backscattered electron detector were used to verify the polycrystalline nature of the produced film. The results revealed that the crystallization process was slower on the polymer substrate, yielding more dispersed grains with sizes ranging from 3 to 7 μm, in contrast to the grains formed directly on the Si substrate, which measured between 1 and 3 μm in diameter. The structural and electromechanical properties of the poly-Si are highly influenced by the annealing conditions and the choice of substrate, underscoring the importance of automation and reproducibility for scalability. Incorporating a post-annealing step in the fabrication process could further address potential process-related defects, enhancing both the device performance and overall repeatability. To assess the thermal stability and piezoresistive response of the polycrystalline film, test devices incorporating piezoresistors were fabricated. The piezoresistors and electrical contacts formed a metal–semiconductor–metal junction that exhibited sensitivity to light. The developed poly-Si demonstrated a temperature coefficient of resistance (TCR) of −2471 ppm/°C, indicating sensitivity to environmental temperature variations. The adaptation of such an alloy as a metal catalyst at lower temperatures broadens the compatibility of these materials with various fabrication processes for devices such as pressure, force, or temperature sensors on both rigid and flexible substrates. When designing piezoresistive sensors, it is crucial to consider factors such as sensor geometry and the design and location of piezoresistors to optimize performance and effectively utilize the material’s sensitivity.

Another work, by Butt et al. [14], is also connected with silicon usage in the field of sensor engineering. According to the authors, a silicon waveguide sensor utilizing germanium on silicon-on-insulator (Ge-on-SOI) technology offers significant advantages for gas detection, specifically in the mid-infrared (MIR) wavelength range. This spectral region is particularly critical for gas sensing because many gases exhibit pronounced absorption features within it. The elevated refractive index of germanium facilitates the effective confinement of light within the waveguide, thereby enhancing light–matter interactions and improving the sensor’s sensitivity to low concentrations of gases. The suspended design of the waveguide effectively minimizes the optical losses by limiting interactions with the substrate, thereby boosting the overall sensing performance. The sensing mechanism is solely based on physical principles (specifically optical absorption), without involving any chemical bonding or adsorption of CO_2_ molecules onto the waveguide surface, which imparts excellent reusability to the sensor. The interaction is non-invasive, ensuring that no lasting alterations or degradation of the waveguide occur during its operational use. Consequently, this proposed structure can be reused indefinitely without any loss in performance, provided that both the waveguide and optical setup are maintained appropriately.

The study by Chen et al. [15] proposed a novel two-core fiber Bragg grating (FBG)-based sensor for high-precision curvature measurements. By inscribing two FBGs within a specially engineered two-core fiber, a high bending sensitivity was achieved across a wide bending range. Additionally, the sensor exhibits compensation for cross-sensitivity to environmental variables such as temperature and humidity, enhancing its suitability for deployment in complex environments. This innovative sensor has the potential to facilitate specialized engineering applications, where bending and curvature measurements are crucial. 

In conclusion, micro- and nanosensors are at the forefront of technological advancements across various fields. The combination of novel fabrication techniques, a wide range of applications, and continuous performance enhancements underscores their importance in driving innovation and addressing global challenges. I would like to take this opportunity to thank all the authors who have submitted their papers to this Special Issue and all the reviewers for their contributions in evaluating the submitted papers and their efforts in improving the quality of the manuscripts. Also, I am very grateful to the *Micromachines* Editorial Board for giving me the opportunity to be a guest editor of this Special Issue.
